# Serum biomarkers of brain injury after uncomplicated cardiac surgery: Secondary analysis from a randomized trial

**DOI:** 10.1111/aas.14033

**Published:** 2022-02-19

**Authors:** Mikael Barbu, Kristján Jónsson, Henrik Zetterberg, Kaj Blennow, Oscar Kolsrud, Sven‐Erik Ricksten, Göran Dellgren, Kerstin Björk, Anders Jeppsson

**Affiliations:** ^1^ Department of Molecular and Clinical Medicine Institute of Medicine Sahlgrenska Academy University of Gothenburg Gothenburg Sweden; ^2^ Department of Cardiology Blekinge Hospital Karlskrona Sweden; ^3^ 56749 Department of Cardiothoracic Surgery Sahlgrenska University Hospital Gothenburg Sweden; ^4^ 56749 Clinical Neurochemistry Laboratory Sahlgrenska University Hospital Mölndal Sweden; ^5^ Department of Psychiatry and Neurochemistry Institute of Neuroscience and Physiology Sahlgrenska Academy University of Gothenburg Mölndal Sweden; ^6^ 4919 Department of Neurodegenerative Disease UCL Institute of Neurology London UK; ^7^ UK Dementia Research Institute at UCL London UK; ^8^ 56749 Department of Cardiothoracic Anesthesia and Intensive Care Sahlgrenska University Hospital Gothenburg Sweden; ^9^ Department of Anesthesiology and Intensive Care Institute of Clinical Sciences University of Gothenburg Gothenburg Sweden

## Abstract

**Background:**

Postoperative cognitive dysfunction is common after cardiac surgery. Postoperative measurements of brain injury biomarkers may identify brain damage and predict cognitive dysfunction. We describe the release patterns of five brain injury markers in serum and plasma after uncomplicated cardiac surgery.

**Methods:**

Sixty‐one elective cardiac surgery patients were randomized to undergo surgery with either a dextran‐based prime or a crystalloid prime. Blood samples were taken immediately before surgery, and 2 and 24 h after surgery. Concentrations of the brain injury biomarkers S100B, glial fibrillary acidic protein (GFAP), tau, neurofilament light (NfL) and neuron‐specific enolase (NSE)) and the blood–brain barrier injury marker β‐trace protein were analyzed. Concentrations of brain injury biomarkers were correlated to patients’ age, operation time, and degree of hemolysis.

**Results:**

No significant difference in brain injury biomarkers was observed between the prime groups. All brain injury biomarkers increased significantly after surgery (tau +456% (25th–75th percentile 327%−702%), NfL +57% (28%−87%), S100B +1145% (783%−2158%), GFAP +17% (−3%−43%), NSE +168% (106%−228%), while β‐trace protein was reduced (−11% (−17−3%). Tau, S100B, and NSE peaked at 2h, NfL and GFAP at 24 h. Postoperative concentrations of brain injury markers correlated to age, operation time, and/or hemolysis.

**Conclusion:**

Uncomplicated cardiac surgery with cardiopulmonary bypass is associated with an increase in serum/plasma levels of all the studied injury markers, without signs of blood–brain barrier injury. The biomarkers differ markedly in their levels of release and time course. Further investigations are required to study associations between perioperative release of biomarkers, postoperative cognitive function and clinical outcome.

**Editorial Comment**

In a secondary analysis of a randomized trial, Barbu and colleagues report that several markers of neuronal and astroglial injury, but not blood–brain barrier injury, increased after uncomplicated elective cardiac surgery with cardiopulmonary bypass in the absence of clinical signs of neurological damage.

## 1 INTRODUCTION

Over the last years, highly sensitive methods to measure brain injury markers in serum or plasma have been developed.^1^ These proteins are released into peripheral blood in response to acute neuronal injury. Blood biomarkers are today routinely used in the care of traumatic brain injury and ischemic stroke, or following successful resuscitation after cardiac arrest.^2^, ^3^, ^4^, ^5^, ^6^, ^7^, ^8^, ^9^ Their release can also correlate with the severity of the brain damage and patient clinical outcome.^4^, ^5^, ^6^, ^7^, ^8^, ^9^

A few studies have reported on serum and plasma brain injury markers in cardiac surgery patients.^10^, ^11^, ^12^ However, there is no evidence for the practical usability of these markers.^13^ Before plasma concentrations of biomarkers can be used to quantify and forecast neurological complications, the release patterns after uncomplicated cardiac operations, as well as potential interactions with perioperative variables, need to be established.

The aim of the present study was to describe the magnitude and time course of serum and plasma concentrations of proteins reflecting glial and neuronal injury after uncomplicated elective cardiac surgery with cardiopulmonary bypass (CPB). To this purpose, we performed a predefined secondary analysis from data collected in a prospective randomized trial.^14^ A secondary aim was to study potential associations between biomarker levels and patients’ age, operation time, and degree of perioperative hemolysis.

## 2 METHODS

### 2.1 Ethics approval

The study was performed in accordance with the Declaration of Helsinki and approved by the Regional Research Ethics Committee in Gothenburg (entry number: T 847–16 Ad 1003–15). Written informed consent was obtained from all subjects. The study was registered at ClinicalTrials.gov prior to enrolment (identifier: NCT02767154).

### 2.2 Patients

The main study included 84 elective cardiac surgery patients treated from May 2016 to July 2017. Inclusion criteria were all patients aged 50–80 years accepted for elective cardiac operations with an expected CPB time of >75 min. In total, 42 patients were randomized to the colloid group and 42 to the crystalloid group. In the present substudy, 22 patients were excluded, 11 in the colloid group and 11 in the crystalloid group, because the research resources to prepare the specific blood samples for analyses of brain injury markers were unavailable, due to completion of surgery after office hours. One patient in the colloid group with postoperative stroke confirmed by computed tomography was also excluded. Hence, 61 patients (74% men, mean age 66.3 ± 7.1 years) were included in this substudy. Patient characteristics are listed in Table 1.

**TABLE 1:** Preoperative and perioperative variables in 61 cardiac surgery patients randomized to colloid (dextran‐40) or crystalloid priming solution. Values are given as mean and standard deviation (SD) or number (%). CABG = coronary artery bypass surgery; CPB = cardiopulmonary bypass.

	All n=61	Colloid prime n=30	Crystalloid prime n=31
Male gender (n, %)	45 (74%)	22 (73%)	23 (74%)
Age (yrs)	66.3 ± 7.1	65.3 ± 6.4	67.3 ± 7.7
Body mass index (kg/m^2^)	26.9 ± 4.0	26.8 ± 4.1	27.0 ± 3.9
Diabetes (n, %)	8 (13%)	4 (13%)	4 (13%)
Operation (n, %)			
CABG	24 (39%)	13 (43%)	11 (35%)
Aortic valve replacement	12 (19%)	7 (23%)	5 (16%)
Mitral valve repair or replacement	10 (16%)	6 (20%)	4 (13%)
Valve+CABG	7 (11%)	1 (3%)	6 (19%)
Other	8 (13%)	3 (10%)	5 (16%)
Euroscore II (%)	2.0 ± 2.3	1.7 ± 1.2	2.3 ± 3.0
Hemolysis index 2 hours postoperatively	32 ± 30	20 ± 13	42 ± 36
CPB time (min)	98 ± 36	90 ± 26	107 ± 42
Aortic clamp time (min)	73 ± 31	66 ± 24	79 ± 37
Total operation time (min)	188 ± 52	185 ± 46	190 ± 57
Mean arterial pressure during CPB (mmHg)	60 ± 8	60 ± 8	59 ± 8
Flow rate during CPB (L/min/m^2^)	2.5 ± 0.1	2.5 ± 0.1	2.4 ± 0.1

### 2.3 Study design

The main study was a prospective, randomized, single‐center, double‐blind controlled study. The patients were randomized 1:1 to either a dextran‐based priming solution (PrimECC, XVIVO Perfusion AB, Gothenburg, Sweden) or a crystalloid‐based priming solution (Ringer acetate (Fresenius Kabi AB, Uppsala, Sweden) and mannitol (Fresenius Kabi AB)). The primary endpoint was oncotic pressure during CPB. The study’s main results have been published elsewhere.^14^

For the present secondary analysis, blood samples for analysis of five brain injury markers (GFAP, S100B, NSE, tau, and NfL) and the blood–brain barrier injury marker β‐trace protein were collected from the arterial line at three time points: before surgery (after induction of anesthesia); 2 h after CPB, and 24 h after CPB. At the same time points, the degree of hemolysis was measured as it is known that hemolysis interacts with the analysis of NSE.^15^ Patient data, type of surgery, CPB time, aortic cross‐clamp time and total operating time were registered for all patients. Concentrations of brain injury markers 2 h postoperatively were correlated to patient age, operation time, and hemolysis. No neurocognitive tests were performed.

### 2.4 Clinical management

Details of the clinical management have been published before.^14^ In short, anesthesia was induced with fentanyl and propofol followed by rocuronium, and maintained with sevoflurane. The CPB circuit was primed with 1,300 mL solution according to the patient’s allocation. Cardiopulmonary bypass flow rate was 2.4 L/min/m^2^ and mean arterial pressure was kept to 50‒70 mmHg throughout the procedures by using vasopressors/vasodilators. Cardiotomy suction was used in all patients. Cell saver processing was used at the surgeon’s discretion. All patients were operated at normothermia or mild hypothermia (34–36°C) and rewarmed to 36°C before weaning CPB.

### 2.5 Laboratory tests

The blood samples (approximately 10 mL) for analysis of brain injury biomarkers and β‐trace protein were stored in vacutainer tubes (Becton Dickinson, Franklin Lakes, NJ, USA) containing ethylenediaminetetraacetic acid (EDTA) (for plasma) and inert polymer gel (for serum). Within 1 h, these tubes were centrifuged at 4°C for 10 min at 4,000 revolutions per minute, and then 500 µL aliquots of plasma and serum, respectively, were pipetted into Eppendorf tubes (Becton Dickinson). Samples were stored at −80°C before being transported to the Clinical Neurochemistry Laboratory for analysis. Intra‐assay coefficients of variation were <10% for all measurements, indicating that it was valid to compare relative changes.

Serum S100B, GFAP, and NSE concentrations were measured on a Modular E170 instrument (Roche Diagnostics, Penzberg, Germany) using reagents from the manufacturer. Plasma tau concentration was measured using a commercial kit developed for the Quanterix single molecule array (SiMoAa) platform (Quanterix, Billerica, MA, USA).^16^ Serum NfL concentration was measured using an in‐house assay on the SiMoA platform (Quanterix).^17^ Beta‐trace protein (prostaglandin D synthase) concentration was measured by nephelometry on the Atellica NEPH 630 System (Siemens Healthineers, Erlangen, Germany). Hemolysis index was measured on a Cobas c 501 instrument (Roche Diagnostics, Penzberg, Germany) by bichromatic spectrophotometry at 570 nm and 600 nm wavelength pair.^18^ The hemolysis index was used to calculate free hemoglobin in g/L using the formula [f‐Hb = (0.915 × hemolysis index +2.634)/100].^18^ Hemolysis was indicated by a free hemoglobin level >0.5 g/L. Samples with hemolysis were excluded from analyses of changes in NSE concentrations over time,^15^ but were included in the correlation analyses.

### 2.6 Statistical analyses

Data were tested for normal distribution using Shapiro–Wilk test. Normally distributed continuous data are presented as mean and standard deviation. Not normally distributed continuous data are presented as median and 25th–75th percentile. Group comparisons were performed with Student’s *t*‐test or Mann–Whitney test. Paired analyses were performed with paired *t*‐test or Wilcoxon’s test. Between‐group differences in variables measured at more than one time point were analyzed with analysis of variance for repeated measurements. Student’s *t*‐test was used to test the differences between groups at the various time points if the analysis of variance indicated a significant time–group interaction. Categorical data are presented as numbers and percentages and compared with Fisher’s exact test. Correlations were analyzed with Spearman’s rank sum test. A *p* < 0.05 was considered to be significant. No sample‐size calculation was performed for this secondary analysis. Sixty‐one observations give a power of >99% with a two‐sided paired t‐test, to detect a mean difference of 10 pg/mL in Tau concentration, with a significance level of 0.05. Due to the skewness of the variable, loge scale of difference in tau concentration was used, corresponding to the assumed mean of 2.3 pg/ml and an observed standard deviation of 0.7. All statistical analyses were performed with Statistica software (StatSoft, Tulsa, OK, USA) or SPSS 25.0 (IBM, Armonk, NY, USA).

## 3 RESULTS

### 3.1 General

A patient flow diagram is included in supplemental materials, Table S1. There were no significant differences in baseline variables between patients operated with colloid and patients operated with crystalloid priming solutions, Table 1. In‐hospital mortality was 3.3%. The remaining patients recovered after surgery and were discharged after a mean of 6.9 days.

### 3.2 Glial injury markers

**FIGURE 1:**
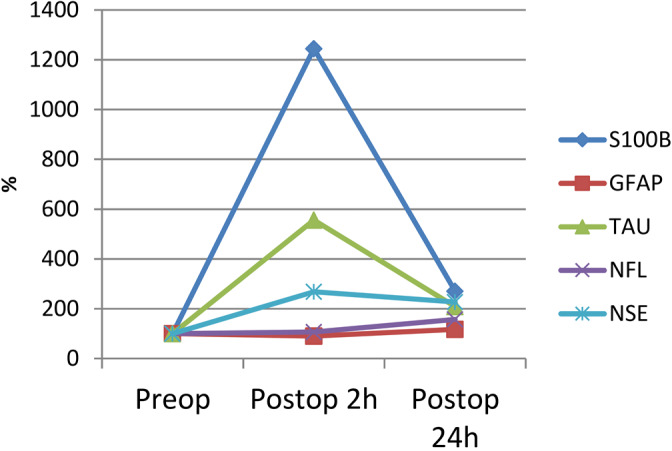
Median percentage changes in plasma/serum concentrations of brain injury markers from baseline to 2h and 24h after cardiac surgery. The baseline level is set to 100%. The variability for changes in biomarkers is presented in Figure 2 and in Table 2. GFAP =glial fibrillary acidic protein; NfL =neurofilament light; NSE =neuron‐specific enolase

**FIGURE 2:**
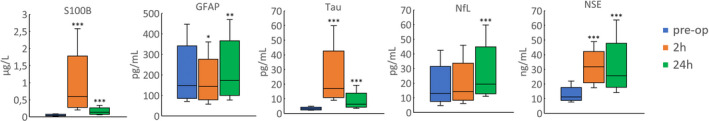
Box plots of concentrations of biomarkers before surgery (blue), 2h after CPB (orange), and 24h after CPB (green). The values are median and 25th and 75th percentiles. Wilcoxon's paired test was used to analyze changes from baseline. CPB =Cardiopulmonary bypass, GFAP =glial fibrillary acidic protein; NfL =neurofilament light; NSE =neuron‐specific enolase. *p < 0.05; **p < 0.01; ***p<0.001 compared with the preoperative concentration

Both S100B and GFAP significantly increased over time in both the colloid and the crystalloid group, without significant intergroup differences (Table 2 and Figure 1). For the two groups combined, the maximum increase from baseline in S100B was markedly higher, +1,145% (25^th^‐75th percentile 783%‐2,158%, *p* < 0.001 compared with baseline levels), than the maximum increase in GFAP, +17% (−3%–43%, *p* = 0.003). The concentration of S100B was higher at 2 h compared with 24 h while the highest GFAP concentration was measured at 24 h (Table 2 and Figure 2). Concentrations of S100B were still elevated 24 h after surgery compared with preoperative values.

**TABLE 2:** Brain injury markers and β‐trace protein in the dextran group and the crystalloid group. Analysis of variance (ANOVA) for repeated measurements was used to indicate between‐group differences. Student’s *t*‐test was used to test the differences between groups at the various time points if the ANOVA indicated a significant time–group interaction. Wilcoxon’s paired test was used to analyze changes from baseline. Median and 25th and 75th percentiles are shown. Neuron‐specific enolase samples with hemolysis (free hemoglobin >0.5 g/L) have been excluded. ANOVA =analysis of variance; GFAP =glial fibrillary acidic protein; NfL =neurofilament light; NSE =neuron‐specific enolase; *p < 0.05; **p < 0.01; ***p < 0.001 vs. preoperative values within the same group

	All patients	Dextran‐based prime	Crystalloid prime	*p*‐value, dextran vs. crystalloid prime (ANOVA for repeated measurements time*group)
	N = 61	N=30	N = 31	
S−100B (µg/L)				
Preop	0.04(0.03–0.07)	0.04(0.03–0.05)	0.05(0.04–0.07)	0.078
Postop 2 h	0.59(0.35–0.98)***	0.55(0.34–0.77)***	0.77(0.34–1.14)***	
Postop 24 h	0.13(0.09–0.18)***	0.12(0.10–0.15)***	0.16(0.09–0.20)***	
GFAP (pg/mL)				
Preop	148(100–235)	154(90–214)	143(102–240)	0.80
Postop 2 h	144(100–192)*	120(99–188)**	157(100–221)*	
Postop 24 h	173(122–262)**	170(117–269)**	179(125–257)**	
NfL (pg/mL)				
Preop	13.1(10.5–20.5)	14.1(10.5–21.3)	12.8(10.2–20.4)	0.86
Postop 2 h	14.3(10.6–21.3)	17.6(12.0–22.3)	12.8(10.3–20.1)	
Postop 24 h	19.3(14.6–30.0)***	20.4(13.6–30.6)***	19.3(14.8–31.0)***	
Tau (pg/mL)				
Preop	3.1(2.6–3.7)	2.9(2.4–3.7)	3.2(2.7–3.7)	0.74
Postop 2 h	16.9(12.8–25.2)***	17.2(11.1–21.7)***	16.6(13.3–26.5)***	
Postop 24 h	6.3(4.9–8.6)***	5.8(4.5–7.4)***	6.9(5.8–8.8)***	
NSE (ng/mL)	N=51	N=29	N=22	
Preop	11.7(9.9–13.2)	12.1(10.2–14.0)	11.2(9.9–12.6)	0.10
Postop 2 h	31.7(24.4–35.5)***	29.6(22.0–35.2)***	34.1(30.1–37.2)***	
Postop 24 h	25.6(21.4–32.1)***	27.5(21.6–33.0)***	24.0(21.3–30.1)***	
Β‐trace protein (mg/L)	N=61	N=30	N=31	
Preop	0.67(0.59–0.76)	0.67(0.59–0.76)	0.67(0.60–0.76)	0.34
Postop 2 h	0.60(0.52–0.72)***	0.61(0.50–0.76)*	0.58(0.52–0.72)**	
Postop 24 h	0.60(0.52–0.73)*	0.64(0.53–0.79)	0.57(0.51–0.73)	

### 3.3 Neuronal injury markers

Tau protein, NfL, and NSE significantly increased over time in both the colloid and the crystalloid group without significant intergroup differences (Table 2 and Figure 1). In ten patients, the NSE results were excluded from further analyses of changes over time because of hemolysis. For the two groups combined, the increase in tau protein from baseline was markedly higher, +456% (25th–75th percentile 327–702%, *p *< 0.001), than the increase in NSE, +168% (106–228%, *p *< 0.001), and NfL, +57% (28–87%, *p *< 0.001). Tau and NSE concentrations were higher 2 h after surgery than after 24 h, while the highest level of NfL was measured 24 h after surgery (Table 2 and Figure 2). Tau and NSE levels were still significantly elevated after 24 h compared with preoperative values.

### 3.4 Blood–brain barrier damage marker

The concentration of β‐trace protein decreased from baseline in all patients both within the colloid and the crystalloid group (Table 2).

### 3.5 Correlations with patient data and intraoperative variables

There were statistically significant positive correlations between patient age and the changes in S100B, GFAP, and NfL at 2 h (Table 3). The CPB time had a positive correlation with changes in S100B, GFAP, tau, and NSE at 2 h. Both S100B and NSE had a significant positive correlation with the level of hemolysis (results for plasma‐free hemoglobin >0.5 g/L not excluded), while such correlations were not observed for the other biomarkers.

**TABLE 3:** Correlation coefficients (Spearman’s rank sum test) and *p*‐values for patient‐related (age) and operative factors (cardiopulmonary bypass (CPB) time and hemolysis) vs. concentrations of brain injury markers 2 h after CPB. GFAP =glial fibrillary acidic protein; NfL =neurofilament light; NSE =neuron‐specific enolase; R = correlation coefficient

	Age	CPB time	Hemolysis
S100B	**R = 0.38**	**R = 0.38**	**R = 0.38**
	***p* = 0.003**	***p* = 0.003**	***p* = 0.003**
GFAP	**R = 0.36**	**R = 0.31**	R = 0.23
	***p* = 0.006**	***p* = 0.017**	*p* = 0.085
Tau	R = 0.16	**R = 0.56**	R = 0.05
	*p* = 0.24	***p*<0.001**	*p* = 0.70
NfL	**R = 0.29**	R = 0.18	R = 0.05
	***p* = 0.027**	*p* = 0.17	*p* = 0.70
NSE	R = 0.07	**R = 0.69**	**R = 0.77**
	*p* = 0.59	***p*<0.001**	***p*<0.001**

Values in bold highlight significant correlations where *p* < 0.05.

## 4 DISCUSSION

Our main finding was that serum and plasma concentrations of all measured biomarkers increased after surgery, despite the absence of any obvious neurological or neurocognitive symptoms, and without group difference between dextran‐based colloid prime and crystalloid prime. The magnitude and the time to the highest measured concentration differed markedly among the biomarkers. Concentrations of the injury markers were moderately correlated to patients’ age, operation time, and/or the degree of hemolysis in the samples.

Among the studied biomarkers, S100B had the largest increase, with a tenfold increase 2 h after surgery. However, the results should be interpreted with caution as S100B is also expressed in extracerebral cell types, such as adipocytes, chondrocytes, and melanocytes.^19^ This may influence plasma concentration and reduce the specificity of the analysis. Increased postoperative concentration of S100B has also been attributed to re‐transfusion of pericardial shed blood.^19^ In the present study, S100B levels correlated moderately with operation time, age, and the degree of hemolysis, which needs to be considered.

Neuron‐specific enolase is a glycolytic enzyme expressed in the cytoplasm of neurons and is highly specific for neuronal damage.^20^ It is also expressed in platelets, red blood cells, and neuroendocrine‐derived tumors.^20^ Serum concentrations of NSE are directly influenced by hemolysis,^21^ which was confirmed in the present study. In fact, simultaneous measurements of NSE in cerebrospinal fluid and serum for patients undergoing surgical aortic valve replacement showed an increase only in serum samples.^22^ Hence, NSE may be less reliable in the early postoperative phase after cardiac surgery, where hemolysis is common.^21^

Tau, NfL, and GFAP have no extracerebral sources and may be more suitable for identifying neurological damage in the cardiac surgery setting. Tau is highly specific for central nervous system damage, with high expression in unmyelinated cortical axons.^23^ Recently, a small study has shown that tau and NfL protein increased after uncomplicated major surgical procedures, such as arthroplasty, performed under general anesthesia.^24^ The rapid release of tau with an early rise in concentration, as illustrated in the present study, in combination with high central nervous system specificity, indicates that tau could potentially become a useful marker for perioperative neurological events in cardiac surgery.

Elevated levels of neurofilament light in cerebrospinal fluid and blood are highly specific for axonal damage.^25^ Available studies on NfL release kinetics indicate peak values varying from 7 to 10 days after ischemic stroke or traumatic brain injury.^26^, ^27^ After resuscitation from cardiac arrest with severe brain hypoxia, serum NfL shows a very marked increase also at early time points (1–3 days).^6^ Hence, NfL seems less suitable for early evaluation in cardiac surgery patients, but later measurements may be important for long‐term prognosis. One recent study highlighted the effects of CPB, reporting higher tau and NfL plasma levels in on‐pump cases compared with when surgery was performed off‐pump.^28^

Glial fibrillary acidic protein is a protein found in the astroglial skeleton, exclusively in the central nervous system.^29^ Among the five biomarkers in the present study, GFAP had the smallest elevation dynamics (+17% at 24 h after CPB compared with baseline). In a study following ischemic stroke patients, serum GFAP values peaked 3–4 days after the initial insult,^30^ which indicates that the present study may have missed the GFAP peak. However, GFAP may be of interest because it is highly specific for brain injury, as no elevated levels are noted in multi‐trauma patients without traumatic brain injury.^31^

The increases of the biomarkers in plasma/serum were observed despite the absence of neurological or neurocognitive symptoms. Besides true neuronal injury, an increase in peripheral brain injury markers can derive from a disrupted blood–brain barrier, allowing equalization of a higher liquor gradient.^32^, ^33^ In the present study, there was no indication of blood–brain barrier disruption after surgery in the time period studied, as β‐trace protein levels were not increased. Beta‐trace protein is considered a reliable marker of blood–brain barrier injury.^34^ Notably, there was a small but statistically significant reduction in β‐trace protein concentration 2 and 24 h after surgery, compared with preoperatively (median relative difference −11%). Most likely this is caused by hemodilution given the fluid administration during CPB.

All brain injury markers correlated with CPB time and/or age. It remains unclear whether this reflects a larger subclinical brain injury in older patients and in patients with longer operation time or if it is unrelated to size of the injury. Furthermore, S100B and NSE concentrations correlated with hemolysis, which causes interference during the early postoperative phase, a potential confounder not observed for the other markers.

The present study has important limitations but also strengths. The results are based on a limited number of subjects in a secondary analysis of a randomized trial, and should therefore be considered as exploratory, rather than conclusive. Twenty‐three (27%) of the original 81 patients in the randomized study were excluded from analysis in this secondary analysis, mostly due to study logistics, which may bias the results. As stated, no specific postoperative neurocognitive examinations or brain imaging investigations were performed. Hence, the study provides no information about associations between neurocognitive function, morphological brain injury, and brain injury biomarkers after cardiac surgery. Further studies are necessary to explore these associations. Strengths include the comparatively large number of brain injury markers evaluated and the standardized sample collection.”

In conclusion, uncomplicated cardiac surgery with CPB is associated with an increase in serum and plasma levels of brain injury biomarkers. The release patterns differ considerably between different biomarkers. The measured concentration levels 2 h after surgery correlated with patient age and CPB time. Further investigations to study associations between perioperative release of biomarkers, postoperative cognitive function, and clinical outcomes are warranted.

## CONFLICTS OF INTEREST

Dr. Zetterberg is a Wallenberg Scholar supported by grants from the Swedish Research Council (#2018‐02532), the European Research Council (#681712), Swedish State Support for Clinical Research (#ALFGBG‐720931), the Alzheimer Drug Discovery Foundation (ADDF), USA (#201809‐2016862), the AD Strategic Fund and the Alzheimer's Association (#ADSF‐21–831376‐C, #ADSF‐21–831381‐C and #ADSF‐21–831377‐C), the Olav Thon Foundation, the Erling‐Persson Family Foundation, Stiftelsen för Gamla Tjänarinnor, Hjärnfonden, Sweden (#FO2019‐0228), the European Union's Horizon 2020 research and innovation program under the Marie Skłodowska‐Curie grant agreement No. 860197 (MIRIADE), and the UK Dementia Research Institute at UCL, London. Dr. Blennow is supported by the Swedish Research Council (#2017‐00915), the Alzheimer Drug Discovery Foundation (ADDF), USA (#RDAPB‐201809‐2016615), the Swedish Alzheimer Foundation (#AF‐742881), Hjärnfonden, Sweden (#FO2017‐0243), the Swedish state under the ALF agreement between the Swedish government and the County Councils (#ALFGBG‐715986), and the European Union Joint Program for Neurodegenerative Disorders (JPND2019‐466‐236). Dr. Jeppsson has received honorarium for consultancy from Xvivo Perfusion. Otherwise, none of the authors report any conflict of interests.
